# Frequent users of the ambulance service in a Swedish region: a retrospective cohort study

**DOI:** 10.1186/s13049-026-01551-2

**Published:** 2026-01-10

**Authors:** Fredrik Alm, Lisa Kurland, Karin Hugelius

**Affiliations:** 1 School of Health Sciences, Faculty of Medicine and Health, Örebro University, S-701 82 Örebro, Sweden; 2 School of Medical Sciences, Faculty of Medicine and Health, Örebro University, Örebro, Sweden; 3 Faculty of Medicine and Health, Örebro University, Örebro, Sweden

**Keywords:** Frequent user, Ambulance services, Emergency medical services, Healthcare systems, Emergency care

## Abstract

**Background:**

This study aimed to describe the characteristics of frequent users of ambulance services and their corresponding ambulance missions.

**Methods:**

A retrospective cohort study using register data from a Swedish region (approximately 308,000 inhabitants) was conducted. The dataset covered ambulance missions dispatched between 2019 and 2023; data from 2020–2022 were analyzed, with the extended timeframe (2019–2023) used to identify frequent users. Frequent users were defined as patients with four or more ambulance missions within a 365-day period, divided into moderate (patients with 4–11 missions within 365 days) and high-frequency users (≥ 12 missions within 365 days). Missions associated with these users were referred to as frequent, moderate-frequent, high-frequency, or non-frequent ambulance missions.

**Results:**

Between 2020 and 2022, 73,461 ambulance missions were utilized by 41,407 unique patients. Of these 41,407 patients, 8.4% were frequent users (7.9% moderate and 0.5% high-frequency users), but accounted for 29.0% of all ambulance missions. The remaining 91.6% were non-frequent users, accounting for 71.0% of the ambulance missions. Frequent users were older than non-frequent users (median age 75 vs 62, *p* < .001). Frequent ambulance missions were more often dispatched for dyspnoea (17.8% vs 11.6%, *p* < .001), convulsions (4.2% vs 2.1%, *p* < .001), and abdominal pain (10.5% vs 8.7%, *p* < .001) compared with non-frequent ambulance missions. Frequent ambulance missions were also more likely to be assigned to a higher triage level, and 77.7% resulted in transport to the emergency department. Compare to moderate-frequent ambulance missions, high-frequency missions were more often dispatched for psychiatric emergencies (6.2% vs 2.1%, *p* < .001) and intoxications (5.3% vs 2.0%, *p* < .001), and more frequently resulted in transport to psychiatric emergency care (3.6% vs 1.4%, *p* < .001), or not being conveyed (24.0% vs 16.1%, *p* < .001).

**Conclusions:**

Frequent ambulance users account for a large proportion of all ambulance missions. The results indicate that frequent users are often in need of emergency care, and understanding this population’s needs is essential to ensure appropriate care. However, the group is heterogeneous and can be divided into frequent and high-frequency users, which have different characteristics. We suggest that future research investigates system-level approaches to identify frequent ambulance users and implement care plans to address patients’ needs and reduce ambulance utilisation.

## Background

A well-functioning ambulance service is fundamental to ensuring public safety [1, 2]. Recent reports show both an increasing demand of ambulance services [3] and longer response times [4]. Ambulance personnel are not only encountering patients with complex medical conditions and comorbidities more often, but they are also seeing an increasing number of patients without preexisting health issues using ambulance services [3]. This increasing demand makes it imperative to understand the experiences and interactions of those who most frequently use the ambulance services—not only because their usage patterns affect overall utilisation, but because they may represent a particularly vulnerable group. Prior research shows that frequent users of emergency services often present with complex health problems and face significant social challenges [5, 6]. They have also been shown to have higher mortality as compared to other patient groups [7].

The lack of a standardised definition of a frequent user of ambulance services complicates comparisons between studies [5, 8]. In the literature, the range of frequent users (or callers) varies from 4–5 times per year to five times per month [9, 10]. The corresponding definition of frequent users of the emergency department has, in prior literature, been suggested to be defined as four or more visits within one year [11]. Moreover, the prevalence of frequent users within emergency medical services has previously been reported to be between 0.2% and 23% [5].

Given their complex needs and substantial impact on ambulance services, frequent users constitute a critical group for targeted research. Knowledge about frequent users of ambulance services in Scandinavia, remains limited [12, 13]. Advancing the understanding of the characteristics and care trajectories of frequent ambulance users is essential for developing tailored interventions that improve patient outcomes and system efficiency. This study aimed to describe the characteristics of frequent users of ambulance services and their corresponding ambulance missions within a Swedish region.

## Methods

### Study design

This was a retrospective cohort study based on register data from the Örebro region in Sweden.

### Study population and setting

The study population comprised patients who used ambulance services in the Örebro region between 2020 and 2022, identified from a dataset of dispatched ambulance missions. Exclusion criteria included secondary transports (e.g., inter-hospital ambulance missions), and ambulance missions lacking a registered personal identification number, reducing the dataset by 10,148 ambulance missions prior to analysis.

The Örebro region comprises urban cities, including a central city with approximately 160,000 residents, hosting a university hospital, two other cities with approximately 30,000 and 23,000 residents, with one smaller hospital each, and rural areas. Calls to the national emergency number 112 are handled by the emergency call centre (SOS Alarm). The patients´ conditions are assessed using a medical dispatch index. An ambulance is dispatched with one of four priorities. Priority 1 represents time-sensitive and potentially life-threatening conditions that require an immediate response, and the ambulance uses lights and sirens. Priority 2 represents an acute but not time-sensitive condition, while Priority 3 is a non-urgent ambulance need. Priority 4 corresponds to assignments where no medical care is required during the ambulance transport.

The ambulance service is part of the regional healthcare. There are 17 ambulances in the Örebro region, each staffed with two personnel, of which at least one is a nurse specialised in prehospital emergency care. Ambulance personnel use the Rapid Emergency Triage and Treatment System (RETTS) to determine the patients’ triage category [14]. The RETTS considers the patients’ chief complaint, including a brief medical history and vital signs. All these parameters are considered to score the urgency of the patient’s condition. Triage level ranges, in five steps, from ‘red’ (indicating a potential life-threatening condition) to green (non-urgent condition) [15].

### Data source

The dataset was compiled from automatically recorded data from the ambulance service’s digital medical records where data from the emergency medical call centre (EMCC) was integrated. Data retrieved from the register included the ambulance call identification number, the patients’ age (at the first index ambulance mission) and sex, dispatch index and priority, and the date and time of the ambulance was dispatched via EMCC. Data from the ambulance service included the triage level according to RETTS and potential referral to care after assessment by ambulance personnel.

### Definitions

The study population was composed of patients with their corresponding ambulance missions between 1 January 2020 and 31 December 2022. The extended dataset of ambulance missions dispatched between 2019 and 2023 was used to classify individuals as frequent or non-frequent users, thereby reducing the risk of misclassification caused by truncated observation windows.

Guided by previous research [5, 10], frequent users were defined as patients with four or more ambulance missions within a 365-day period, subdivided into moderate (4–11 missions within 365 days) and high-frequency users (≥ 12 missions within 365 days). Non-frequent users were defined as patients with 1–3 missions within 365 days.

An ambulance mission was defined as an emergency ambulance dispatched from the EMCC. Ambulance missions were classified based on the user involved (as defined above): missions involving frequent users were reffered to as frequent ambulance missions and futher divided into moderate- or high-frequency ambulance missions, while missions involving non-frequent users were reffered to as non-frequent ambulance missions. 

The EMCC dispatch index, i.e., the reason for the ambulance being dispatched according to EMCC, was condensed into broader categories to simplify analysis (Table 1). Care after the initial ambulance service assessment was classified as ‘emergency department’, ‘outpatient clinic’, ‘inpatient ward’, ‘psychiatry clinic’, ‘primary care centre’, ‘non-conveyance’, or ‘others’.

**Table 1:** Examples of how the dispatch index, i.e. the reason for the ambulance being dispatched according to EMCC, were grouped into categories

Category	Dispatch index
Chest pain	Chest pain
	Chest pain Adult
	Chest pain/Heart Disease
	Chest pains/Heart disease
Headache/dizziness	Headache/Dizziness
	Dizziness
	Dizziness Children
	Dizziness Adult
	Head Pain
	Head Pain Adult
	Head Pain Children
Non-specific chief complaints	Suspected Need for Medical Care
	Unclear Care Needs
	Unclear Care Needs Children 1–6 years
	Unclear Care Needs Infant
	Unclear Care Needs Adult
	Uncertain Information
	Impaired General Condition
	Impaired General Condition Adult
	Altered Behavior
Diabetes/blood sugar abnormalities	Diabetes
	High/Low Blood Sugar
	High/Low Blood Sugar Children
	High/Low Blood Sugar Adul

### Statistical analysis

Continuous variables are presented as medians with interquartile ranges (Q1–Q3). Categorical variables are reported as count (*n*) and per cent (%). Data is organised by non-frequent users and frequent users, with the latter subdivided into Moderate and High. Group comparisons were consistently performed between non-frequent and frequent, and within the frequent group between moderate and high. For comparisons between two groups, the chi-square test was used for categorical/nominal data, an independent t-test for continuous data, and the Mann–Whitney U test for ordinal data. Effect sizes are reported using Phi coefficient. All tests were two-sided, and *p* < 0.01 was considered significant. The statistical analyses were conducted using IBM SPSS Statistics version 28.0.0.0 (IBM, 2021).

### Ethics

The study was approved by the Swedish Ethical Review Authority (Ref. Number: 2024-02794-01).

## Results

### Demographics

**Fig. 1:**
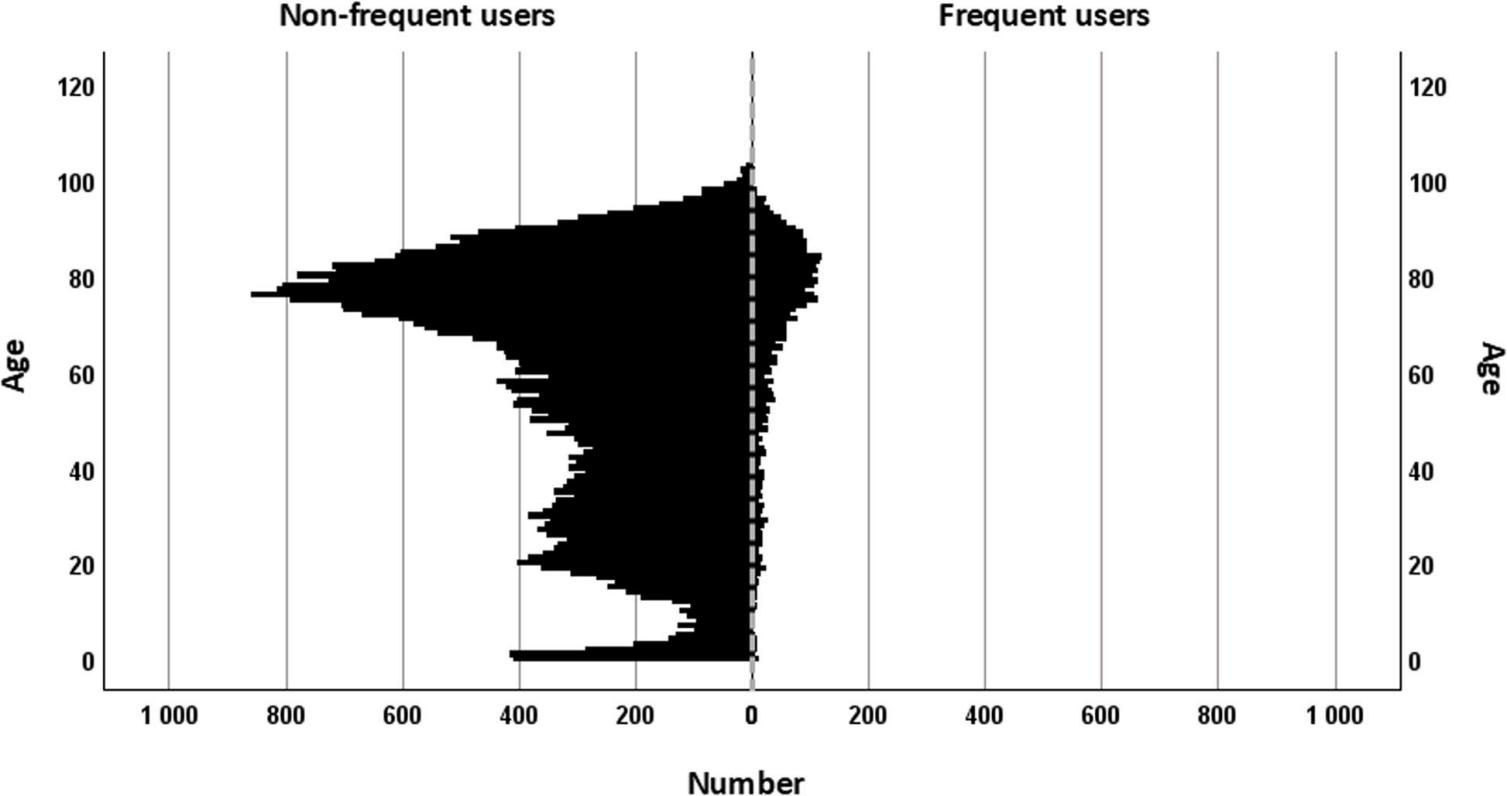
Age distribution among non-frequent and frequent users

Between January 1, 2020, and December 31, 2022, the dataset included 73,461 ambulance missions corresponding to 41,407 unique patients. The sex distribution among these patients was approximately equal (male: 49.2%), and the median age was 64 years (Q1 = 36; Q3 = 78). Overall, 3,473 (8.4%) were classified as frequent users (7.9% moderate, 0.5% high frequency), whereas 37,934 (91.6%) were non-frequent users. Compared to non-frequent users, frequent users were older (median 75 vs 62 years, *p* < 0.001) and more likely to be men (51.5% vs 49.0%, *p* = 0.004; Table 2, and Fig. 1).

**Table 2:** Age and sex among non-frequent users and frequent users

	**Non-frequent users**	**Frequent users**			Non-frequent **vs** Frequent	Moderate **vs** High
		Total	Moderate	High		
	*n* = 37 934	*n* = 3 473	*n* = 3 267	*n* = 206	*p*-value	*p*-value
**Age**						
Median (Q1; Q3)	62.0 (35;78)	75.0 (60;83)	76 (61;84)	64.0 (44;76)	<.001	<.001
**Sex**						
Male *n* (%)	18 584 (49.0)	1 789 (51.5)	1677 (51.3)	112 (54.4)	.004	.398
Female *n* (%)	19 350 (51.0)	1 684 (48.5)	1590 (48.7)	94 (45.6)

### Ambulance missions

Overall, the most common ambulance missions were in the categories of trauma/injuries, chest pain, dyspnoea, and abdominal pain. Frequent ambulance missions were more often dispatched for dyspnoea (17.8% vs 11.6%, *p* < 0.001), convulsions (4.2% vs 2.1%, *p* < 0.001), and abdominal pain (10.5% vs 8.7%, *p* < 0.001) than non-frequent ambulance missions. High-frequency ambulance missions were more commonly dispatched for psychiatric emergencies (6.2% vs 2.1%, *p* < 0.001) and intoxications (5.3% vs 2.0%, *p* < 0.001) compared to moderate-frequent ambulance missions (see Table 3). No significant differences were observed in EMCC dispatch priority between ambulance missions associated with non-frequent and frequent users. Frequent ambulance missions were assigned significantly higher triage levels (RETTS) by the ambulance crew compared to non-frequent missions, whereas high-frequency missions were associated with the lowest triage levels. No other clinically relevant differences in EMCC dispatch priority or time of day (holiday vs weekday) were observed between the groups (Tables 4 and 5).

**Table 3:** Number and proportion of ambulance missions associated with non-frequent and frequent users, by EMCC dispatch category

	**Non-Frequent missions**	**Frequent missions**			Non-frequent **vs** Frequent	Moderate **vs** High
		Total	Moderate	High		
	*n* (%)	*n* (%)	*n* (%)	*n* (%)	*p*-value	*p*-value
Trauma/injuries	10 302 (19.9)	2 373 (11.2)	2 057 (12.2)	316 (7.3)	<.001^b^	<.001^a^
Chest pain	7 009 (13.5)	2 878 (13.6)	2 292 (13.6)	586 (13.6)	.769^a^	.986^a^
Dyspnoea	5 995 (11.6)	3 764 (17.8)	2 957 (17.5)	807 (18.7)	<.001^a^	.073^a^
Abdominal pain	4 535 (8.7)	2 220 (10.5)	1 615 (9.6)	605 (14.0)	<.001^a^	<.001^a^
Non-specific chief complaints	4 354 (8.4)	1 979 (9.3)	1 702 (10.1)	277 (6.4)	<.001^a^	<.001^a^
Stroke	3 940 (7.6)	1 335 (6.3)	1 149 (6.8)	186 (4.3)	<.001^a^	<.001^a^
Headache/dizziness	2 751 (5.3)	863 (4.1)	672 (4.0)	191 (4.4)	<.001^a^	.190^a^
Altered level of consciousness	2 245 (4.3)	838 (4.0)	692 (4.1)	146 (3.4)	.024^a^	.030^a^
Infection/fever	1 852 (3.6)	886 (4.2)	799 (4.7)	87 (2.0)	<.001^a^	<.001^a^
Pain	1 883 (3.6)	623 (2.9)	517 (3.1)	106 (2.5)	<.001^a^	.035^a^
Convulsions	1 082 (2.1)	891 (4.2)	657 (3.9)	234 (5.4)	<.001^a^	<.001^a^
Intoxication	943 (1.8)	564 (2.7)	334 (2.0)	230 (5.3)	<.001^a^	<.001^a^
Bleeding	1 016 (2.0)	418 (2.0)	343 (2.0)	75 (1.7)	.889^a^	.213^a^
Psychiatric emergency	820 (1.6)	623 (2.9)	355 (2.1)	268 (6.2)	<.001^a^	<.001^a^
Other reasons	951 (1.8)	303 (1.4)	225 (1.3)	78 (1.8)	<.001^a^	.019^a^
Diabetes/blood sugar abnormalities	462 (0.9)	292 (1.4)	220 (1.3)	72 (1.7)	<.001^a^	.067^a^
Allergy	557 (1.1)	58 (0.3)	44 (0.3)	14 (0.3)	<.001^a^	.476^a^
Arrythmia	338 (0.7)	152 (0.7)	131 (0.8)	21 (0.5)	.319^a^	.044^a^
Obstetrics	393 (0.8)	39 (0.2)	38 (0.2)	1 (< 0.1)	<.001^a^	.006^a^
Cardiac arrest	300 (0.6)	62 (0.3)	52 (0.3)	10 (0.2)	<.001^a^	.406^a^
Hypo- or hyperthermia	112 (0.2)	42 (0.2)	31 (0.2)	11 (0.3)	.639^a^	.349^a^
Missing value	329	89	72	17		
**Sum:**	**52 169**	**21 292**	**16 954**	**4 338**		

Chi-square test

^a^Phi coefficient < 0.10

^b^Phi coefficient 0.10–0.29

**Table 4:** Priority among ambulance missions associated with Non-Frequent and Frequent users

	**Non-Frequent missions**	**Frequent missions**			Non-Frequent **vs** Frequent	Moderate **vs** High
		Total	Moderate	High		
	*n* (%)	*n* (%)	*n* (%)	*n* (%)	*p*-value^a^	*p*-value^a^
**EMCC dispatch priority**						
1	24 615 (47.3)	9 961 (46.9)	7 834 (46.3)	2 127 (49.1)	.036	<.001
2	24 516 (47.1)	9 922 (46.7)	7 944 (47.0)	1 978 (45.7)		
≥ 3	2 880 (5.5)	1 364 (6.4)	1 138 (6.7)	226 (5.2)		
Missing	158	45	38	7		
**RETTS**						
Red	7 195 (13.9)	3 061 (14.4)	2 578 (15.3)	483 (11.2)	<.001	<.001
Orange	15 353 (29.6)	6 550 (30.9)	5 430 (32.2)	1 120 (25.9)		
Yellow	19 623 (37.9)	8 103 (38.2)	6 279 (37.2)	1 824 (42.2)		
Green	9 440 (18.2)	3 406 (16.1)	2 525 (15.0)	881 (20.4)		
Blue	212 (0.4)	75 (0.5)	63 (0.4)	12 (0.3)		
Missing	346	97	79	18		
**Transport priority**						
1	6 787 (16.4)	2 804 (15.9)	2 361 (16.6)	443 (13.2)	.006	<.001
2	19 601 (47.3)	8 192 (46.6)	6 778 (47.6)	1 414 (42.2)		
≥ 3	15 014 (36.3)	6 584 (37.5)	5 088 (35.7)	1 496 (44.7)		
Missing^b^	10 767	3712	2727	985		

*RETTS *Rapid Emergency Triage and Treatment System

^a^Mann–Whitney U Test

^b^Missing include cases of non-conveyed

**Table 5:** Time points for Ambulance Missions Associated with Non-Frequent and Frequent Users

	**Non-frequent missions**	**Frequent missions**			Non-Frequent **vs.** Frequent	Moderate **vs.** High
		Total	Moderate	High		
	n (%)	n (%)	n (%)	n (%)	*p*-value	*p*-value
Weekdays	36 457 (69.9)	15 089 (70.9)	12 024 (70.9)	3065 (70.7)	.008^a^	.730^a^
Weekend	15 712 (30.1)	6203 (29.1)	4930 (29.1)	1273 (29.3)		

Chi-square test

^a^Effect size (phi coefficient) < 0.10

In total, 75.5% of ambulance missions resulted in transport to EDs, making it the most common destination for both frequent and non-frequent missions (77.7% and 73.9% respectively). This was also the case for moderate and high-frequency ambulance missions (79.6% and 70.4% respectively). High-frequency ambulance missions more often resulted in transport to psychiatric emergency care (3.6% vs 1.4%, *p* < 0.001), or non-conveyance (24.0% vs 16.1%,* p* < 0.001) as compared to missions associated with moderate-frequent users (Table 6).

**Table 6:** Ambulance destination after assessment by ambulance personnel including non- conveyed

	**Non-frequent missions**	**Frequent missions**			Non-Frequent **vs.** Frequent	Moderate **vs.** High
		Total	Moderate	High		
	n (%)	n (%)	n (%)	n (%)	*p*-value	*p*-value
Emergency departments	38 565 (73.9)	16 553 (77.7)	13 500 (79.6)	3053 (70.4)	<.001^a^	<.001^a^
Non-conveyed	10 912 (20.9)	3864 (18.1)	2821 (16.1)	1043 (24.0)	<.001^a^	<.001
Other	1104 (2.1)	146 (0.7)	138 (0.8)	8 (0.2)	<.001^a^	<.001^a^
Psychiatry	420 (0.8)	388 (1.8)	231 (1.4)	157 (3.6)	<.001^a^	<.001^a^
Wards	616 (1.2)	192 (0.9)	131 (0.8)	61 (1.4)	.003^a^	<.001^a^
Outpatient clinics	485 (0.9)	123 (0.6)	112 (0.7)	11 (0.3)	<.001^a^	.004^a^
Primary health care centres	61 (0.1)	22 (0.1)	17 (0.1)	5 (0.1)	.658^a^	.577^a^
Missing	6	4	4	0		

Chi-square test

^a^Phi coefficient < 0.10

## Discussion

The results indicate that frequent users represent a substantial proportion of the total mission volume in the studied region and period. Frequent users were generally older, and their ambulance missions differed in dispatch reasons and were more often assigned higher triage levels compared to non-frequent users. Subgroup analysis revealed that high-frequency users had a different profile compared to moderate and non-frequent users, with their ambulance missions being dispatched more often to psychiatric conditions, assessed as having lower acuity and more frequently not being conveyed.

The results of this study revealed that frequent users were particularly prominent in dispatch categories such as dyspnoea, convulsions, and abdominal pain. In contrast, among high-frequency users, psychiatric conditions and related issues such as intoxication were more prevalent reasons for ambulance contact. These results align with previous findings showing that frequent callers to the emergency call number had complex needs, including chronic physical and mental health conditions, as well as unmet personal, social, and care-related needs [16]. Furthermore, a prior study with a low threshold for frequent use, more than two missions within a 12-month period, demonstrated an association with chronic health problems, mental disorders, and alcohol abuse [13]. Social needs and loneliness have also been suggested as significant reasons for individuals to call for an ambulance among frequent users [17]. However, in the current study, data on social determinants, such as living arrangements, socioeconomic status, and family situations, were not available. These factors are of great interest and should be included in future research to better understand and confirm such relationships.

Previous research has demonstrated that individuals who frequently call emergency ambulance services often perceive their needs as urgent [6]. This perception was generally confirmed by ambulance personnel in this study, who often assessed frequent users’ ambulance missions as urgent. Conversely, this was not the case for high-frequency users, who had their ambulance missions more often assessed as having lower acuity.

Alternative methods of organising medical care for frequent users of emergency care have been suggested [13]. One such suggestion is to flag frequent callers and direct them to other medical services, such as the primary care or multidisciplinary teams [18]. However, the current study indicates that the frequent user often had a genuine need for emergency medical care. This raises concerns about the potential risks of flagging individuals solely based on call frequency, as it may fail to identify acute medical needs. At the same time, the subgroup of high-frequency users was more often assigned a lower triage level by the ambulance personnel, more frequently non-conveyed, and more frequently had mental health issues. This indicates that healthcare services other than ambulance services or the emergency department may be better suited to some of these patients’ needs.

Previous research has shown that some frequent users of emergency care lack other established points of contact within the healthcare system and, therefore, rely on emergency care [19]. Given these insights, it may be beneficial for ambulance services to implement systems that automatically identify high-frequency users with the aim of targeting interventions such as personalised care plans or assessment by mobile teams. Such systems have also been suggested to contribute to identifying patients in need of medical attention after post-discharge after surgery [20]

To build on this understanding, future research should further explore the social networks, medical care contacts, and help-seeking behaviours of frequent ambulance users in comparison to non-frequent users. This would provide valuable insights into how healthcare services can better support individuals with complex or recurring medical needs, ultimately improving care coordination and outcomes across the system. Importantly, this study highlights heterogeneity among frequent users, whose characteristics and needs vary depending on the definition of frequent use applied. While the commonly used threshold of four or more ambulance missions within 365 days offers a practical measure, it may not fully capture the complexity of this population. Our results indicate that high-frequency users often exhibit different patterns and likely different needs compared to moderate users, highlighting the importance of revisiting current definitions. We suggest that future research aim to refine these thresholds to establish clinically relevant criteria for identifying frequent users within ambulance services.

## Strengths and limitations

To our knowledge, this is the first study to describe frequent users of ambulance services in Sweden and one of few studies to describe this population in Scandinavia. However, the study has several limitations. The most significant limitation is the inability to account for varying exposure time, as data on deaths and migration were unavailable. Individuals with shorter observation periods, such as those who died or migrated, may have been misclassified. Additionally, babies born during the end of the study period or individuals who have recently developed a need for frequent care may not have been classified as frequent users, as they may have accounted for less than four ambulance missions during the data period. This risk was mitigated, though not eliminated, by extending the observation window. Consequently, the prevalence of frequent users reported in the current study may still be underestimated. The study focused on patients and ambulance missions but did not include calls to the emergency medical call centre, so-called frequent callers. In general, it is important to note that different studies use different terminology and definitions for frequent users/callers, which can influence the interpretation and comparison of results. Finally, the descriptive approach of the analysis and the presentation of aggregated data at group level may be prone to bias; however, the large and comprehensive population included in the study provides a robust basis for interpretation.

## Conclusions

Frequent ambulance users account for a large proportion of all ambulance missions. The results indicate that frequent users are often in need of emergency care, and understanding this population’s needs is essential to ensure appropriate care. However, the group is heterogeneous and can be divided into frequent and high-frequency users, which have different characteristics. We suggest that future research investigates system-level approaches to identify frequent ambulance users and implement care plans to address patients’ needs and reduce ambulance utilisation.

## Data Availability

The datasets generated and analysed in this study are not publicly available due to Swedish legislation on research ethics and the approval from the Swedish Ethical Review Authority, since the data included personal data.

## Abbreviations

EMCC: Emergency medical call centre
ED: Emergency department
RETTS: Rapid Emergency Triage and Treatment System
